# Case report: Malignant melanoma of the lower limb with gastric metastasis

**DOI:** 10.3389/fonc.2023.1181728

**Published:** 2023-04-11

**Authors:** Qiang Hu, Fengru Zhou, Yuanshui Sun

**Affiliations:** Department of General Surgery, Tongde Hospital of Zhejiang Province, Hangzhou, China

**Keywords:** malignant melanoma, gastric metastasis, tumor, lower limb, case report

## Abstract

**Introduction:**

Malignant melanoma with gastric metastasis is extremely rare. We report a case of gastric metastasis caused by malignant melanoma of the lower limb.

**Case presentation:**

A 60-year-old woman was hospitalized for left plantar pain. The patient found a black maculopapular eruption on the left sole of her left foot, which caused pain when pressed, and the pain was aggravated by walking, so she went to our hospital for treatment. On the second day of admission, the lesion of the left foot was removed under local anesthesia, and the removed tissue was sent for pathological examination. Combined with immunohistochemistry, it was consistent with malignant melanoma. During hospitalization, the patient developed abdominal pain and asked for gastroscopy. Gastroscopy revealed two 0.5 cm × 0.6 cm spots that can be seen arising from the stomach mucosa which were slightly swollen, slightly black in the center, and without erosion, and no abnormality was found in the other parts. At the same time, a biopsy was taken under a gastroscope and pathology suggests malignant melanoma. The patient could not undergo subsequent treatment due to cost. The patient was followed up until February 2022 and was within the survival period.

**Conclusion:**

Malignant melanoma gastric metastasis is extremely rare. When a patient has a previous history of melanoma surgery, this needs to be considered when gastrointestinal symptoms are present, and regular endoscopic screening is recommended. Early surgical treatment and postoperative chemotherapy or combined targeted therapy may improve the prognosis of patients.

## Introduction

Malignant melanoma (MM) is a common malignant tumor in the limbs, which is highly invasive, often occurs as metastatic cancer, and has a very poor prognosis (1). A study shows that the overall survival rate at 2 years was only 4% (2). The common metastatic sites of MM are the inguinal, lungs, liver, and brain, which can also metastasize to the gastrointestinal tract (3). The small intestine is the common metastatic site of MM in the gastrointestinal tract, and metastasis to the stomach is rare (4). Gastrointestinal metastases often delay the diagnosis due to the lack of specific symptoms and signs, and most MM transferred to the stomach is detected at autopsy (5). Reggiani et al. (2) found that 56% of MM had gastrointestinal metastasis, so gastrointestinal metastasis of MM needs great attention. We present this 60-year-old woman with MM to remind us that we need to pay attention to MM with gastric metastasis in clinical work and reduce unnecessary medical disputes.

## Case presentation

**Figure 1:**
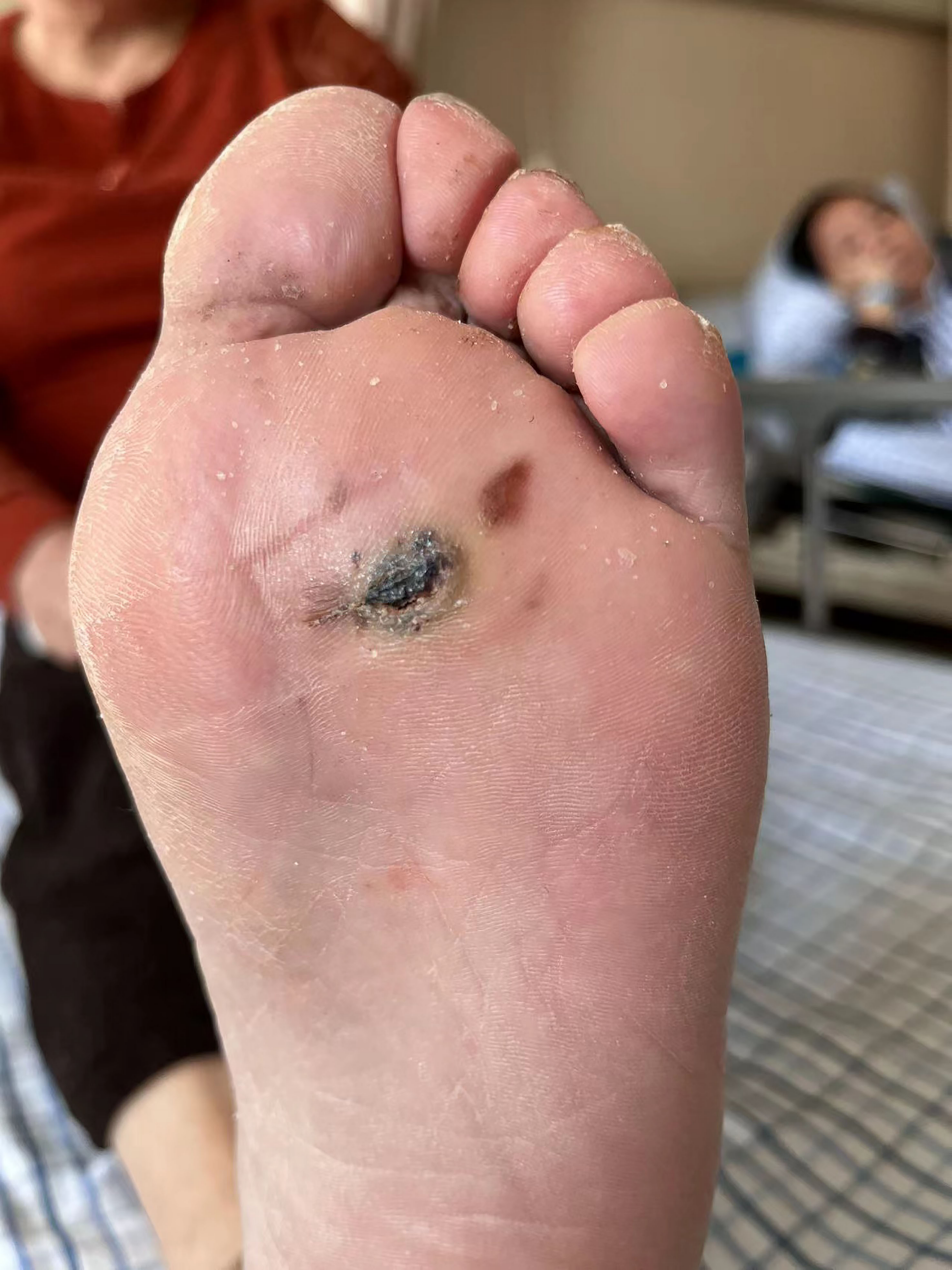
The visible size of the black tumor on the left plantar was approximately 2 × 1 cm.

A 60-year-old woman was admitted to our hospital with left plantar pain. The patient found a black maculopapular rash on the left foot with pressing pain, and the pain was aggravated by walking, so she was hospitalized for further treatment. On physical examination, the visible size of the black tumor on the left plantar was approximately 2 × 1 cm, with an outward expansive growth and tough texture, which cannot be pushed and does not touch the blood vessel (**Figure 1**). Based on the patient’s medical history, she was previously healthy.

**Figure 2:**
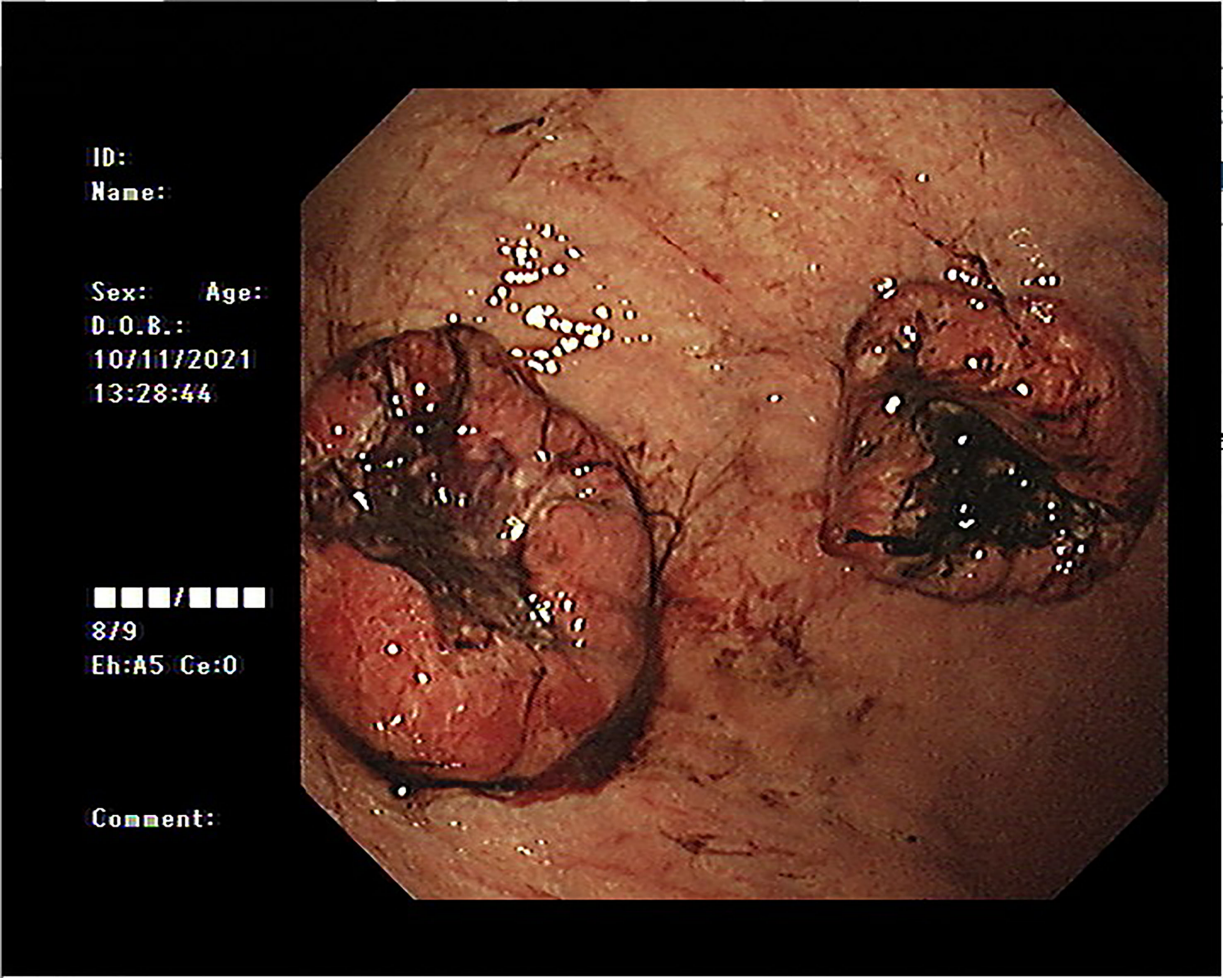
Gastroscopy revealed two 0.5 cm × 0.6 cm spots that can be seen arising from the stomach mucosa which were slightly swollen and slightly black in the center.

**Figure 3:**
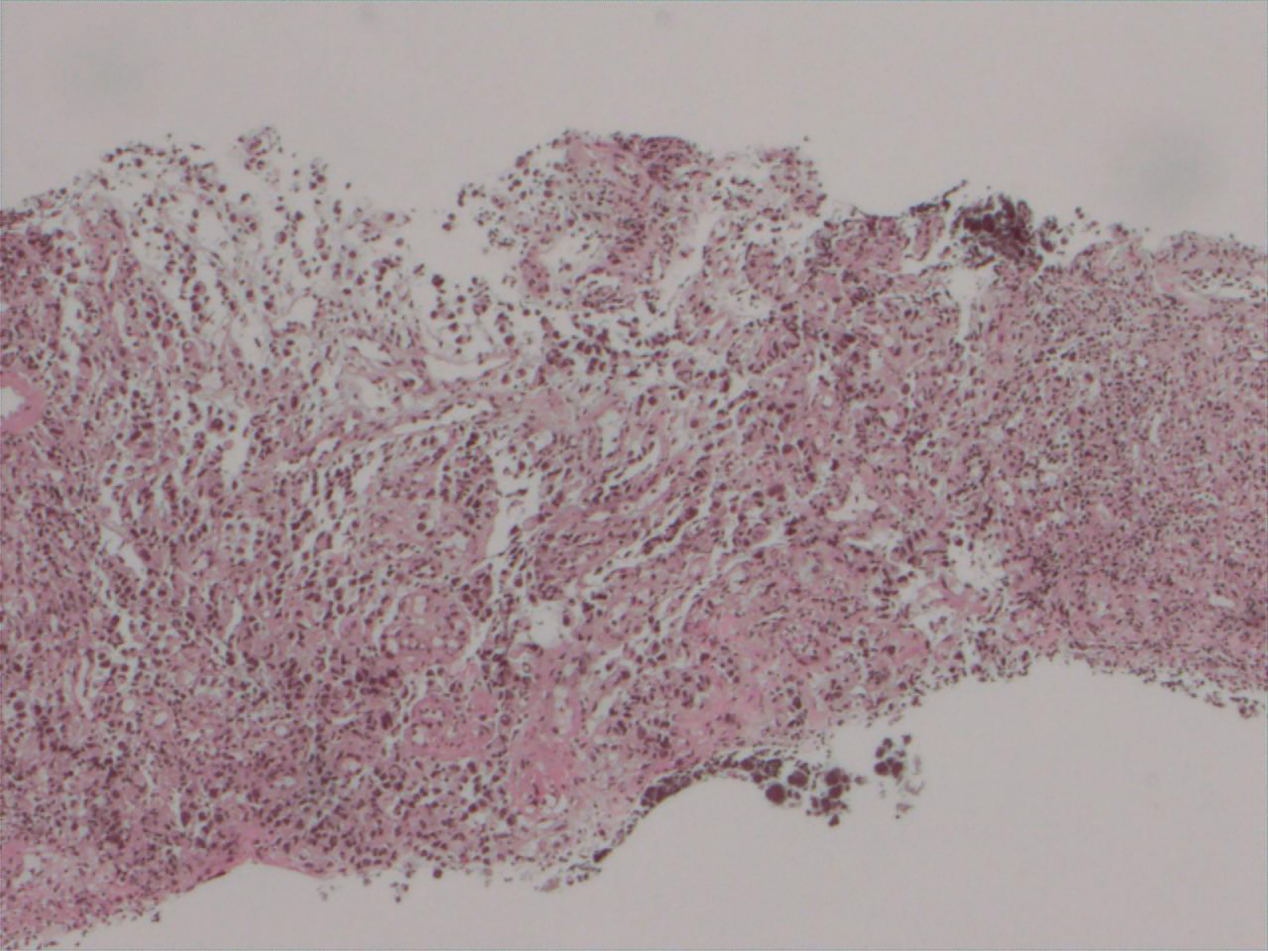
The pathological findings showed that there were nests of abnormal cells, accompanied by pigmentation (H&E, ×10).

**Figure 4:**
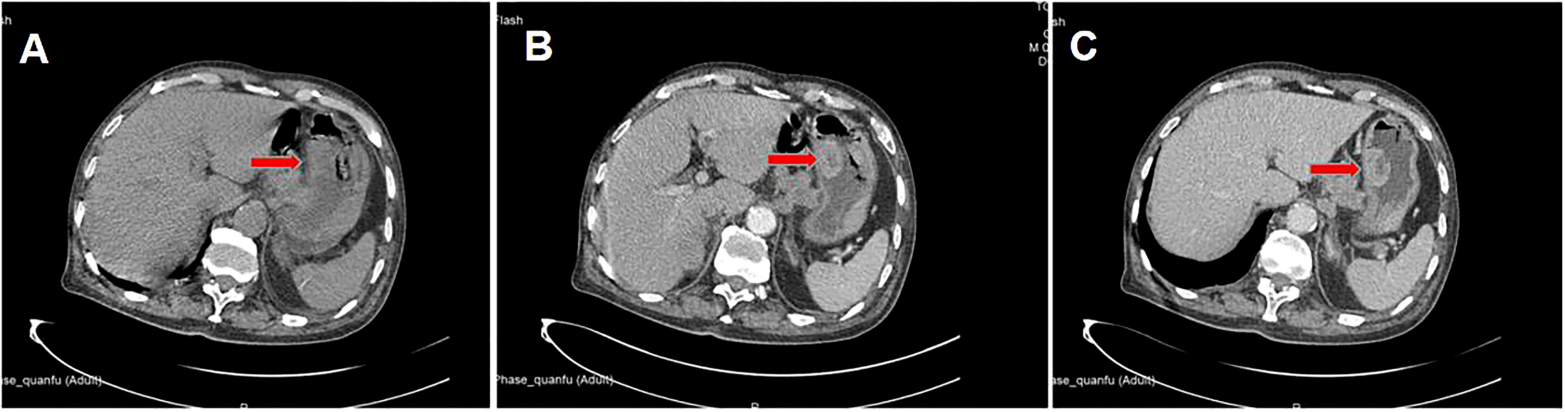
Location of the stomach tumor in abdominal enhanced CT. **(A)** Plain scan; **(B)** arterial phase; **(C)** venous phase.

Because the patient has a strong desire to undergo operation, on the second day after admission, the lesion of the left foot was removed under local anesthesia, and the removed tissue was sent for pathological examination. The pathological findings showed that there were nests of abnormal cells in the epidermis and subcutaneous tissue, accompanied by pigmentation. The results of immunohistochemistry were as follows: PCK (−), HMB45 (+), S-100 (+), P53 (+), Ki-67 (+), CyclinD1 (+), Bcl-2 (+), and P16 (+). Combined with immunohistochemistry, it was consistent with MM. During hospitalization, the patient developed abdominal pain and asked for gastroscopy. Gastroscopy revealed two 0.5 cm × 0.6 cm spots that can be seen arising from the stomach mucosa which were slightly swollen, slightly black in the center, and without erosion, and no abnormality was found in the other parts (**Figure 2**). At the same time, a biopsy was taken under a gastroscope. Pathology suggests MM (**Figure 3**). Abdominal enhanced computed tomography (CT) indicates space-occupying lesions in the gastric body, and malignant tumors were considered (**Figure 4**). No obvious abnormality was found on chest CT. Due to cost, the patient refused further treatment and was discharged automatically. The patient was followed up until February 2022 and she was well until that period of time.

## Discussion

MM is clinically a common malignancy originating from the skin and mucosal tissues and is highly malignant (6). Although the incidence rate of melanoma in China is lower than that in Europe and the United States, it has shown a rapid growth trend in recent years (7). MM has the characteristics of high malignancy, high mortality, poor prognosis, and being prone to distant metastasis. It was reported in the literature that the 2-year survival rate of advanced metastatic malignant melanoma was 15%, the 5-year survival rate was 5%, and the median survival time was only 7.5 months (8). Malignant melanoma mostly occurs in the head and neck, accounting for approximately 50%, and in the gastrointestinal tract and urinary tract each accounting for approximately 25% (9). Melanoma metastasis in the gastrointestinal tract was more common in the small intestine (50%), colon (31.3%), and rectum (25%) and rarely in the stomach (10).

Generally, patients with metastatic MM of the stomach have atypical clinical symptoms (11), including abdominal distension, acid reflux, and other symptoms, as well as complications such as bleeding and perforation (12). They are easy to be confused with various benign and malignant diseases of the digestive tract, resulting in missed diagnosis and misdiagnosis and missing the best treatment period (13). Therefore, if the patient has a history of surgical resection of “melanoma” in the past, finds black tumors in the skin and mucosa during physical examination, and is hospitalized due to digestive system symptoms, MM should be highly suspected.

From the perspective of pathological diagnosis, the diagnosis of gastric metastatic MM is extremely rare clinically, and it has various pathological microscopic manifestations, which are really challenging (14). For microscopic diffuse heteroid cells, in addition to the primary poorly differentiated gastrointestinal carcinoma, we should consider small cell carcinoma, neuroendocrine carcinoma, lymphoma, rare myeloid cell sarcoma, and MM (15). Immunohistochemical examination was helpful for differentiation; for example, the diagnostic specificity and sensitivity of the immunohistochemical index HMB45 for malignant melanoma were 100% and 93%, and the S-100 protein has a strong response to oligopigmented or amelanotic malignant melanoma (16).

In patients with malignant melanoma, patients with metastasis are often in the late stage of the tumor, and the median survival time of patients with metastasis or recurrence is less than 10 months (17). At present, the preferred treatment for melanoma is still surgical resection of all primary lesions and resectable metastases. The study of Ollila et al. (18) found that gastrointestinal metastatic malignant melanoma has a median survival period ranging from 5.4 to 48.9 months after undergoing surgery. High-dose and high-risk patients can be simultaneously assisted in high-dose interferon α-2b (19). It is generally believed that melanoma is not very sensitive to radiotherapy, but it is still a special treatment method in some special cases, which is mainly used for lymph node dissection and postoperative complementary treatment of some head and neck melanoma (especially at the nasal cavity) (20). For MM and metastatic MM that cannot be surgically removed, chemotherapy and targeted treatment can be selected. In recent years, due to the in-depth study of oncogenes and signaling pathways in the pathogenesis and development of melanoma, targeted therapy has become a hot spot, with targeted drugs for genes and signaling pathways, such as BRAF, MEK, and ERK inhibitors (21). The literature shows that regardless of BRAF/NRAS status, small molecule mitochondrial uncoupling agents such as SR4 and niclosamide may become the first-line drugs for the treatment of melanoma and can also be used as adjuvant therapy for patients with failed MAPK inhibitors. In addition, there is also literature indicating that melatonin can serve as one of the drugs to improve the prognosis of melanoma (22).

## Conclusion

MM gastric metastasis is extremely rare. When MM is considered for diagnosis, endoscopic screening should be recommended. Early surgical treatment, postoperative chemotherapy, or combined targeted therapy may improve the prognosis of patients with MM.

## Data availability statement

The datasets presented in this study can be found in online repositories. The names of the repository/repositories and accession number(s) can be found in the article/supplementary material.

## Ethics statement

The studies involving human participants were reviewed and approved by the Tongde Hospital of Zhejiang Province. The patients/participants provided their written informed consent to participate in this study. Written informed consent was obtained from the individual(s) for the publication of any potentially identifiable images or data included in this article.

## Author contributions

QH designed this study. FZ collected the information and images. QH wrote the manuscript. YS reviewed the manuscript. All authors contributed to the article and approved the submitted version.
